# No evidence of drug-induced pancreatitis in rats treated with exenatide for 13 weeks

**DOI:** 10.1111/dom.12040

**Published:** 2012-12-07

**Authors:** K Tatarkiewicz, P Belanger, G Gu, D Parkes, D Roy

**Affiliations:** Amylin Pharmaceuticals, LLCSan Diego, CA, USA

**Keywords:** exenatide, exocrine pancreas, ZDF rat

## Abstract

**Aims:**

The potential association of glucagon-like peptide receptor agonists (GLP-1RAs) with the development of pancreatitis or pancreatic malignancies in patients with diabetes has been suggested. This study evaluated the long-term effects of the GLP-1RA exenatide on pancreatic exocrine structure and function in the Zucker diabetic fatty (ZDF) rat model of type 2 diabetes.

**Methods:**

Rats received subcutaneous twice-daily injections of 0 (control), 6, 40 and 250 µg/kg/day exenatide for 3 months. Clinical signs, body and pancreas weight, food consumption, HbA1c, fasting serum amylase, lipase, glucose and insulin concentrations were evaluated during treatment and after a 28-day off-drug period to assess the reversibility of any observed effects. Morphometric analysis of pancreatic ductal cell proliferation and apoptosis were performed.

**Results:**

Plasma exenatide concentrations were several-fold higher than therapeutic levels observed in humans. No exenatide-related effects were observed on clinical signs, lipase concentration, pancreatic weight, pancreatic histology, ductal cell proliferation or apoptosis. Exenatide improved animal survival, physical condition, glucose concentrations and HbA1c, decreased food intake, and increased serum insulin concentration. Total amylase concentrations, although within normal ranges, were slightly higher in exenatide-treated rats; following the off-drug period, total amylase concentrations were comparable in treated and untreated rats. Exenatide-related minimal-to-moderate islet hypertrophy was observed at doses ≥6 µg/kg/day, with dose-related increases in incidence and degree. These changes were still present after the off-drug period.

**Conclusions:**

Chronic administration of exenatide in ZDF rats resulted in the expected metabolic benefits and improved animal survival, with no adverse effects noted on pancreatic exocrine structure and function.

## Introduction

Glucagon-like peptide-1 receptor agonists (GLP-1RAs) represent a novel therapeutic class for the treatment of type 2 diabetes 1,2 and current treatment guidelines recommend their use after metformin monotherapy fails to lower HbA1c to targets levels 3. Like GLP-1RAs, dipeptidyl peptidase-4 (DPP-4) inhibitors are incretin-based therapies, but they are administered orally. As their introduction into the marketplace, an association with pancreatitis has been suggested for GLP-1RAs and DPP-4 inhibitors, although such cases have been rare 4–8. Analysis of the FDA Adverse Event Reporting System (AERS) database has raised concerns about a potential increase in pancreatitis with long-term use of GLP-1RA-based therapies 9. However, the authors of that paper – aware of limitations of the incomplete and biased character of AERS – concluded that their analysis of the FDA database did not establish that pancreatitis is caused by GLP-1RAs-based therapy 9. Type 2 diabetes and obesity themselves are risk factors for the development of pancreatitis 10 and obesity increases pancreatitis intensity and mortality rate 11, further complicating the assessment of the potential association between GLP-1RA therapy and occurrence of pancreatitis in patients with diabetes.

Investigation of the association between GLP-1RA use and the development of potential adverse pancreatic effects has been studied in several nonclinical studies with conflicting findings. Nonclinical safety and mechanistic studies on exenatide twice daily and exenatide once weekly conducted in rodents, dogs and primates using different routes of administration (e.g. intravenous, subcutaneous) and varying durations (e.g. single dose to chronic, 2-year dosing) did not show pancreatitis based on tests of pancreatic function or histomorphology. Exenatide use in normal, obese and diabetic models did not exacerbate experimental pancreatitis; conversely, it had beneficial actions in some models 12,13. More recent studies also failed to show any adverse effects on the exocrine pancreas related to exenatide or liraglutide (another GLP1-RA) use 14,15. In contrast, in two other studies using normal rats, subtle but significant histological changes along with slight increases in lipase but not amylase concentrations were described in one study 16, and increased ductal cell proliferation without changes in pancreatic enzymes was described in another study 17. Whether GLP-1 receptors are expressed in rodent or human acinar or ductal cells also remains controversial 17–20; therefore, it cannot be excluded that GLP-1 receptor agonism might directly modulate the function of these cell populations.

It was hypothesized that potential deleterious effects of GLP-1 agonism on the exocrine pancreas might be best observed in diabetic animals, rather than healthy animals. The present study was conducted to determine the potential chronic effects of exenatide exposure on exocrine pancreatic structure and function in the Zucker diabetic fatty (ZDF) rat.

## Materials and Methods

### Animals

The study was carried out by an independent Contract Research Organization in compliance with Good Laboratory Practice regulations (21CFR58) and in accordance with applicable laws regarding animal use in research. The ZDF rat was selected as the test system in this study as it is an inbred rat model that, through genetic mutation and a managed diet, closely mimics human adult-onset type 2 diabetes and related complications associated with insulin resistance and β-cell failure.

Male ZDF rats (ZDF/Crl-*Lep^fa^*; Charles River Kingston, Stone Ridge, NY, USA) were housed individually (stainless steel wire-mesh floor cages, automatic watering valve, temperature 19–25 °C, humidity 30–70%, 12:12-hour light cycle) and acclimated for at least 21 days prior to treatment, which started when animals were 8 weeks old. Animals were fed *ad libitum* commercial laboratory diet (Purina Certified Rodent 5008 irradiated, PMI Nutrition International, LLC, Richmond, IN, USA), except when overnight fasting was required for blood sample collection.

### Experimental Design

This study was performed as part of post-marketing request for exenatide twice daily and the study design was reviewed by the United States Food and Drug Administration. Animals were randomly assigned to treatment groups using a computer-based randomization based on pretreatment amylase values (Table 1).

**Table 1:** Study design

	Nominal dose (BID SC injections)		Nominal dose concentration	Dose volume	Number of animals (males)		
				
Group number/treatment	(µg/kg/dose)	(µg/kg/day)	(µg/ml)	(ml/kg/dose)	Main	Recovery	PK
1/Vehicle	0	0	0	0.5	15	8	3
2/Exenatide	3	6	10	0.3	15	8	9
3/Exenatide	20	40	40	0.5	15	8	9
4/Exenatide	125	250	250	0.5	15	8	9

Recovery animals were a subset of each treatment group. PK animals were used for pharmacokinetic evaluation only and were euthanized following their last blood collection. BID, twice daily; SC, subcutaneous; PK, pharmacokinetics.

Exenatide for injection (Amylin Pharmaceuticals, LLC, San Diego, CA, USA), which contains exenatide in diluents (purity of 98.5%) was stored protected from light at 2–8 °C prior to use. Sodium chloride for injection (0.9%, USP; Baxter Healthcare Corporation, Deerfield, IL, USA) was used as the vehicle control. Samples of exenatide formulations were collected on days 1, 22 and 50 and evaluated for exenatide concentration and stability under conditions of use using a validated size-exclusion high-performance liquid chromatography (SEC-HPLC) method.

Animals were treated twice daily by subcutaneous injection at approximately the same time each day for 91 consecutive days. After completion of the treatment phase, a subset of animals from each group was maintained, untreated, for a 28-day recovery period.

### Clinical Observations

Animals were observed twice daily for signs of ill health and/or reaction to treatment and mortality throughout the study. A detailed examination was performed weekly beginning 2 weeks after arrival and throughout the treatment and recovery periods. Individual body weights were measured for all animals within 3 days of arrival, prior to randomization, and weekly (including day 1 and prior to scheduled necropsy). Individual food consumption (excluding animals used for PK analyses) was measured weekly throughout the treatment and recovery periods.

### Clinical Chemistry

Animals were fasted overnight before scheduled blood sample collection. Blood for serum glucose, insulin, amylase, lipase and whole blood HbA1c was collected from anaesthetized animals by jugular or tail venipuncture at baseline and during the dosing and recovery periods on days 30, 60 and 90. Serum glucose, amylase, lipase and whole blood HbA1c were measured according to assay protocols (Modular Analytics, Roche Diagnostics, Indianapolis, IN). Serum insulin was assessed with insulin ELISA (LINCO Research Inc., Saint Charles, MO, USA).

### Pharmacokinetics

Blood samples for plasma exenatide concentration were collected on days 1 and 91 from a subsets of animals from groups 2–4, inclusively, within 15 min of dosing and after the first daily dose thereafter, including the following time points: 5, 15 and 30 min and 1, 2, 4, 6 and 8 h (within 15 min of the second dose). Samples were also collected from a subset of animals from group 1 at a single time point 15 min after the first daily dose on days 1 and 91. Samples were stored at −80 °C for further analyses using a validated ELISA (Intertek dba ALTA Analytical Laboratory, San Diego, CA, USA) 21. Pharmacokinetic parameters were estimated using WinNonlin pharmacokinetic software version 5.2.1 (Pharsight Corp., Mountain View, CA, USA). A non-compartmental approach consistent with the subcutaneous injection route of administration was used for estimation of *C*_max_ and area-under-the-curve (AUC).

### Gross Pathology

Surviving main-study animals were euthanized on day 92 and surviving recovery animals were euthanized on day 120. Animals were exsanguinated from the abdominal aorta after isoflurane anaesthesia. Blood samples were obtained, and pancreata were collected, examined, weighed and fixed in 10% neutral buffered formalin for histologic analysis. Necropsy examinations were conducted under the supervision of a board-certified veterinary pathologist. Pancreas:body weight ratio (using the terminal fasted body weight obtained at necropsy) was calculated.

### Histological Evaluation of Pancreas

The pancreata were embedded in paraffin within 48 h following necropsy. Five-micron-thick sections from the body of the pancreas were cut at four different levels with approximately 100 µm between each level. Two sections (one each from 1st and 3rd level) from each animal were stained with haematoxylin and eosin (H&E). The histopathologic evaluation was performed by a board-certified veterinary pathologist and peer-reviewed. Sections adjacent to H&E-stained sections obtained from each level were processed to detect ductal cell proliferation and apoptosis with a Ki-67 mouse monoclonal antibody (diluted at 1:15, Dako, Carpinteria, CA, USA) and a TUNEL staining kit (Apoptag peroxidase kit, Millipore S-7100, Billerica, MA, USA), respectively. Each section was dually stained with a cytokeratin-20 (CK-20) mouse monoclonal antibody (diluted at 1:25, Dako) to identify pancreatic ductal cells. Diaminobenzidine (Dako) and Vector VIP substrate (Vector Labs, Burlingame, CA, USA) were used as chromogens.

Quantification of proliferation and apoptosis were conducted by counting a targeted total of 1000 to 2000 CK-20-positive ductal cells (centroacinar cells and epithelial cells lining the intercalated, intralobular and interlobular ducts) per animal, using at least two slides for each marker. Only those ductal cells with clear nuclei were counted with Image-Pro Plus software (version 6.3; Bethesda, MD, USA). For each animal, the proportion of Ki-67-positive cells was calculated by dividing the Ki-67 cell count by the total ductal cell count. Similar calculations were conducted with TUNEL positive ductal cells.

### Statistical Analyses

Data are presented as mean ± SD and/or incidence. For each endpoint, the treatment groups were compared to the controls. Levene's test was used to assess homogeneity of group variances. When Levene's test was not significant (p ≥ 0.01), a pooled estimate of the variance [mean square error (MSE)] was computed from a one-way analysis of variance (anova) and utilized by a Dunnett's comparison of each treatment group with the control group. When Levene's test was significant (p < 0.01), comparisons with the control group were made using Welch's *t*-test with a Bonferroni correction. Results of all pair-wise comparisons were reported at 0.05 significance levels. Comparison of survival curves was performed using the log-rank Mantel-Cox test. Statistical tests comparing percentage proliferation and apoptosis in each treatment group to the control group were performed using two-sided contrasts.

## Results

### Dosing Formulation Analysis

Exenatide was not detected in samples collected from group 1 (vehicle) formulations. All samples collected from dose formulations were within ±10% of their nominal concentrations with occasional small deviations up to ±15%, which did not affect the scientific validity of the study.

### Clinical Observations

Clinical signs noted in controls that were judged to be attributed to the progression of type 2 diabetes disease state (typically observed in this animal model) included decreased activity, generalized weakness, thinness/prominent backbone, eyes partly closed, cold to the touch, dehydration, abnormal breathing or abnormal breathing sounds. Additional clinical signs were also noted along the tail (severed tail, discolouration of the skin with or without lesions/scabs/discharge) and on the prepuce (swelling, skin red/white sometimes accompanied with lesions with or without mucoid/white/green discharge). Owing to the extent and severity of some of the above clinical signs, five control animals required veterinary treatment or progressed such that the animals required humane euthanasia. Animals administered exenatide exhibited fewer clinical signs of disease progression than did control animals. Exenatide-treated animals were observed to have dorsal skin scabs or dry skin, with increased incidence in 250 µg/kg/day-treated rats (group 4) compared to controls; this effect was considered exenatide-related, but not adverse. There were no other exenatide-related clinical observations at any dose level.

### Body Weight and Food Intake

**Figure 1:**
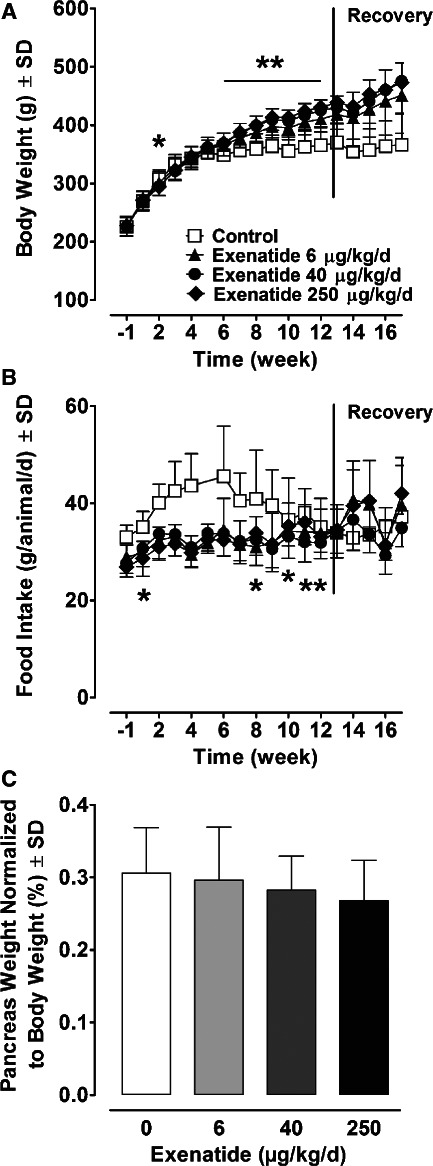
Time course of (A) body weight and (B) food intake changes in ZDF rats dosed subcutaneously with twice daily exenatide (6, 40 and 250 µg/kg/day) or vehicle injections for 3 months followed by a 1-month recovery period without treatment. *p < 0.05 exenatide at 40 and 250 µg/kg/day, **p < 0.05 all exenatide groups, respectively, versus vehicle control. (C) Terminal pancreatic weight normalized to body weight, *p < 0.05 all exenatide groups versus vehicle control.

Group mean body weight and weight gain in control animals were consistent with the anticipated disease model. At approximately week 4 of the treatment period, mean body weight for control animals stabilized while that of exenatide-treated rats showed a continuous, dose-related increase that persisted during the recovery period (figure 1A). Overall weight gain from predose baseline during the 12-week treatment period was 84.8, 90.7 and 92.7% in animals treated with exenatide at 6, 40 and 250 µg/kg/day, respectively, compared with 64.3% in the control group. During the recovery period, mean weight gain was more evident in treated animals, as control animals maintained lower weight gain or exhibited weight loss.

Mean food intake of animals administered exenatide was lower than that of control animals overall, which was inversely correlated with increases seen in body weight in exenatide-treated rats (figure 1B); this effect was an expected pharmacological effect of exenatide. Differences in food intake between control and treatment animals were more noticeable from weeks 1 to 10 of treatment and returned to control levels thereafter and during the recovery period. In control animals, increases in food consumption did not correlate to increases in mean body weight, which was reflective of the disease state.

### Glycaemic Control

**Figure 2:**
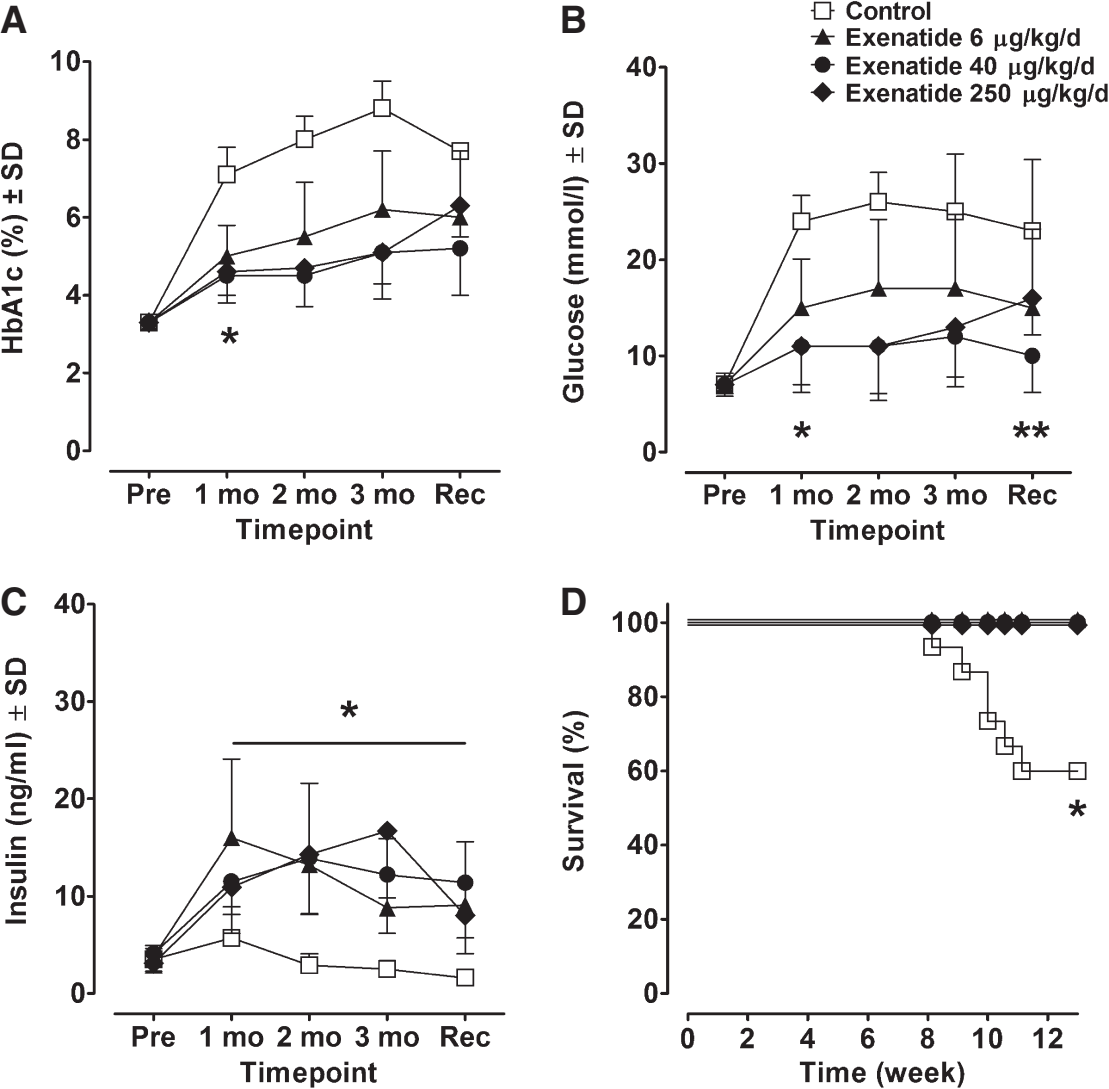
Time course of (A) HbA1c and (B) serum fasting glucose in ZDF rats dosed subcutaneously with twice daily exenatide (6, 40 and 250 µg/kg/day) or vehicle injections for 3 months followed by a 1-month recovery period without treatment. *p < 0.05 all exenatide groups, **p < 0.05 exenatide at 6 and 40 µg/kg/day, respectively, versus vehicle control. (C) Time course of serum fasting insulin in diabetic ZDF rats dosed subcutaneously with twice daily exenatide (6, 40 and 250 µg/kg/day) or vehicle injections for 3 months followed by a 1-month recovery period without treatment. *p < 0.05 all exenatide groups. (D) Exenatide improved survival of ZDF rats dosed subcutaneously twice daily at 6, 40 and 250 µg/kg/day for 3 months in comparison to control untreated group, *p < 0.001.

Mean HbA1c values in controls increased on day 30 and remained elevated throughout the treatment and recovery periods (figure 2A). Mean HbA1c levels in exenatide-treated groups throughout the treatment and recovery periods were lower than those observed for control animals and were higher than baseline levels. The change from baseline was the smallest at 40 and 250 µg/kg/day exenatide doses and was lower than that seen in controls. Changes in HbA1c values throughout the study concurred with trends observed in changes in glucose concentrations. Mean glucose concentrations in controls increased more than threefold on day 30 compared to baseline and remained elevated throughout the treatment and recovery periods (figure 2B). Mean glucose concentrations for exenatide-treated rats were lower than those observed in control animals and increased by 1.6- to 2-fold above that of pre-treatment levels. Differences between control and exenatide-treated animals were most notable at 40 and 250 µg/kg/day exenatide dose levels and were still apparent at the end of the recovery period.

In control animals, mean insulin values remained comparable to baseline values throughout the treatment and recovery periods (figure 2C). In exenatide-treated animals, mean insulin concentrations increased significantly by 2.8- to 4.2-fold on day 30 and remained elevated throughout the treatment and recovery periods compared to baseline and control animals. Observed changes in HbA1c, glucose and insulin in treatment groups were expected pharmacological effects of exenatide.

### Animal Survival

Exenatide-treated animals showed significantly improved survival, which was attributed to an overall improvement in general physical condition and health (figure 2D). Five control animals were euthanized during the treatment period and one control animal was euthanized during the recovery period due to generalized deterioration of the animals' health status. The cause of the poor health status of these control animals was established to be secondary complications due to their diabetic state. Two control animals were also found dead during the study period and the exact cause of death could not be established. One exenatide-treated (250 µg/kg/day) animal was found dead at end of the recovery period shortly after blood collection. The exact cause of death could not be determined from macroscopic and microscopic examinations but potential contributing factors included weight loss, decreased food consumption and blood collection procedure via venipuncture. As this death occurred during the off-drug period and in the absence of any prior clinical signs, a relationship to exenatide was considered unlikely.

### Pancreatic Function and Structure

**Figure 3:**
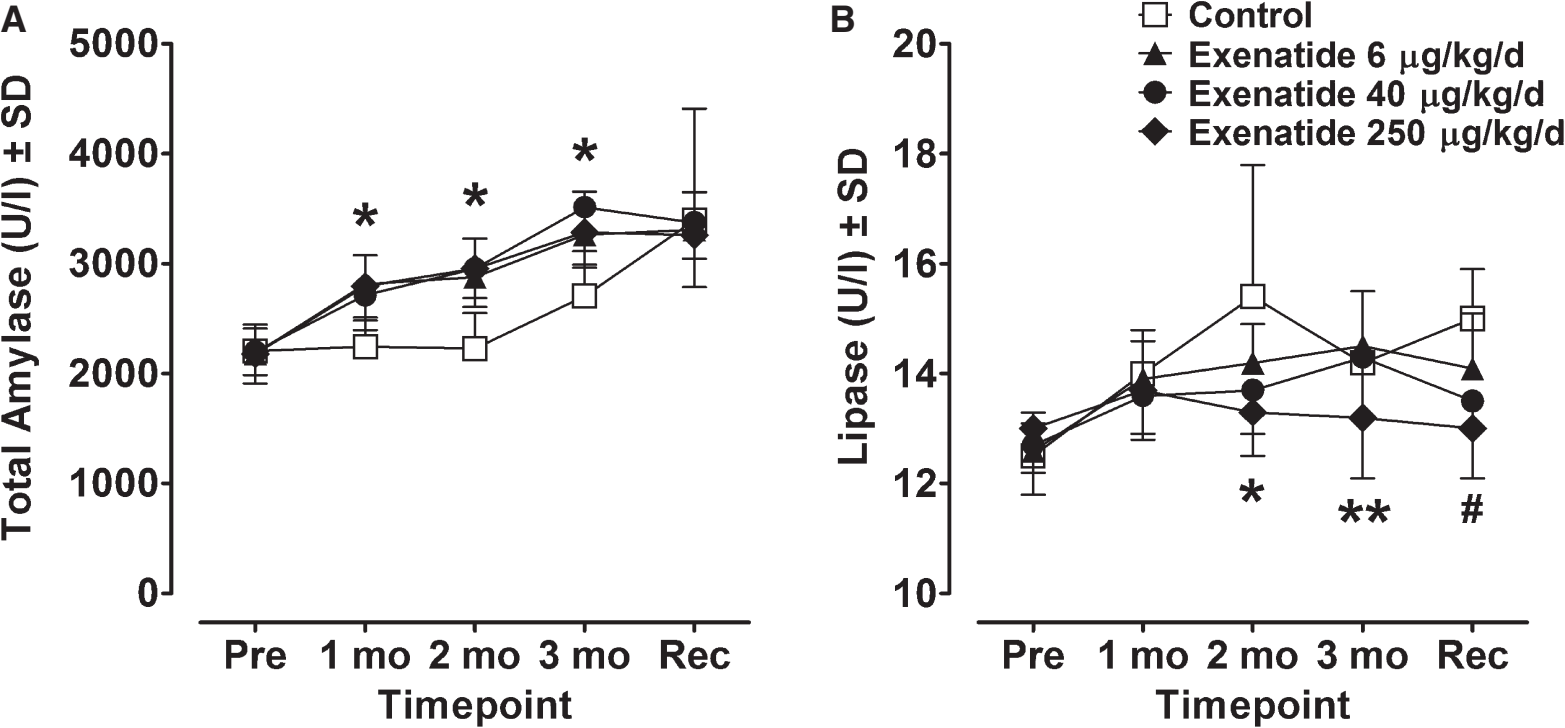
Effects of exenatide on (A) serum fasting amylase and (B) lipase in ZDF rats dosed subcutaneously twice daily at 6, 40 and 250 µg/kg/day or with vehicle for 3 months followed by a 1-month recovery period without treatment. *p < 0.05 all exenatide groups, **p < 0.05 exenatide at 250 µg/kg/day, #p < 0.05 exenatide at 40 and 250, respectively, versus vehicle control.

Mean total amylase concentrations in controls remained comparable to baseline on days 30 and 60 and increased to 3395 ± 1016 U/l on day 90. Mild and time-dependent increases in mean total amylase concentrations, reaching statistical significance in comparison to controls, were seen in all exenatide-treated groups during the treatment phase (figure 3A). In exenatide-treated rats, mean amylase concentration reached a plateau during the treatment period (observed on the last sampling day, day 90) and remained stable until the end of the recovery period. Of note, these minor changes in amylase concentrations were within the same range of values seen in controls at the end of the recovery period and were well below the changes that are typically observed during experimentally induced pancreatitis. There was a slight increase in mean lipase concentrations on day 30 in all groups (including controls) compared to baseline values. The increase remained stable at all time points in the exenatide-treated groups but increased slightly in controls. On days 60 and 90 and at the end of recovery, mean lipase concentrations were lower in exenatide-treated groups than in the control group; these changes were dose-dependent (figure 3B).

There were no exenatide-related effects on absolute pancreas weight or pancreas weight relative to body weight (figure 1C). No exenatide-related macroscopic changes in pancreata from main and recovery necropsies were found. A dose-related increase in the incidence and degree (minimal to moderate) of islet hypertrophy was observed during the recovery period with exenatide doses of ≥6 µg/kg/day (Table 2). No adverse effects of exenatide on the exocrine pancreas (acinar and ductal cells) were observed across all exenatide-treated groups. Incidental microscopic findings, including acinar cell focal hyperplasia or necrosis, duct intraluminal concretion and subacute or acute inflammation were observed in the pancreas across all groups including controls and were not considered toxicologically significant based on incidence, severity, distribution and historical control data (Table 2).

**Table 2:** Incidence and severity of histological changes in pancreas

	Exenatide dose (µg/kg/day)							
	
Parameter	0 (n = 16)	6 (n = 15)	40 (n = 15)	250 (n = 15)	0 (n = 7)	6 (n = 8)	40 (n = 8)	250 (n = 8)
*Treatment phase*	*Main*				*Recovery*			
Islet hypertrophy (total)	0	7	12	13	0	5	7	7
Minimal	0	6	11	10	0	4	5	7
Slight	0	1	1	2	0	1	2	0
Moderate	0	0	0	1	0	0	0	0
Hyperplasia acinar cell: focal (total)	1	1	2	1	0	0	2	1
Minimal	0	1	1	1	0	0	1	1
Slight	1	0	1	0	0	0	1	0
Acinar cell regeneration (total)	0	0	0	0	0	1	0	0
Slight	0	0	0	0	0	1	0	0
Acinar cell necrosis (total)	0	0	0	0	1	0	0	0
Slight	0	0	0	0	1	0	0	0
Duct intraluminal concretion (total)	2	3	4	5	0	2	1	0
Minimal	1	2	4	4	0	1	1	0
Slight	0	1	0	1	0	1	0	0
Moderate	1	0	0	0	0	0	0	0
Subacute inflammation (total)	0	0	0	1	0	0	1	0
Slight	0	0	0	1	0	0	1	0
Acute inflammation (total)	3	1	0	0	1	0	0	1
Minimal	1	1	0	0	1	0	0	1
Slight	1	0	0	0	0	0	0	0
Moderate	1	0	0	0	0	0	0	0
Vascular/perivascular inflammation (total)	0	0	0	0	1	0	0	0
Slight	0	0	0	0	1	0	0	0
Haemorrhage (total)	0	0	0	0	1	0	0	0
Slight	0	0	0	0	1	0	0	0

### Pharmacokinetics (PK)

**Figure 4:**
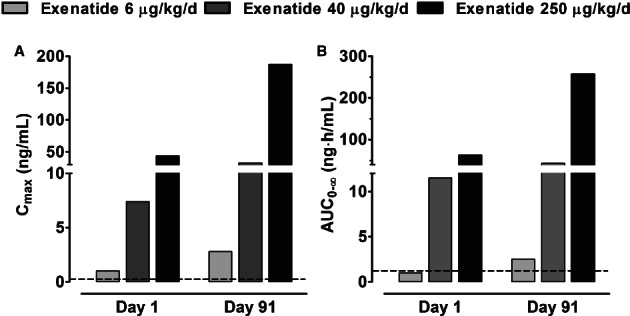
Plasma exenatide exposure in ZDF rats dosed subcutaneously twice daily at 6, 40 and 250 µg/kg/day or with vehicle for 3 months. (A) Peak plasma exenatide concentrations (*C*_max_) and (B) calculated AUC_[0–∞]_. Dotted horizontal lines represent mean values observed in patients with type 2 diabetes mellitus treated with the standard exenatide regimen: *C*_max_ = 0.211 ng/ml and AUC_[0–∞]_ = 1.036 ng/h/ml.

Peak plasma exenatide concentrations were reached within 30 min post-dose for all groups, followed by a mono-exponential decline which captured the terminal elimination phase for all profiles. The half-life of the first daily dose was estimated to be between 0.4 and 0.9 h. Exposure [*C*_max_ (figure 4A) and AUC_[0-∞]_ (figure 4B)] increased substantially after 3 months of exenatide administration with accumulation ratios between 2.7 and 4.4, despite pre-dose concentrations below the lower limit of quantification (23 pg/ml) on day 90. Exposure increased with ascending exenatide dose level and was proportional between 20 and 125 µg/kg/dose (dose levels of 40 and 250 µg/kg/day, respectively) on days 1 and 91 but was greater than proportional to dose level between 3 and 20 µg/kg/dose (6 and 40 µg/kg/day, respectively).

### Histomorphometry

**Figure 5:**
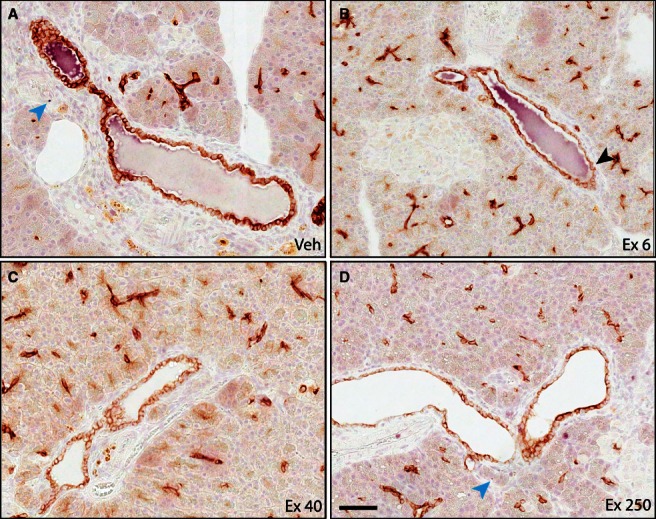
Ki-67 expression in the pancreatic ductal cells was examined after vehicle (A) or exenatide 6 µg/kg/day (B), 40 µg/kg/day (C) or 250 µg/kg/day (D) treatments. Ki-67-positively stained nuclei (black arrow head) were rarely seen in the pancreatic ductal cells, identified via immunocytochemical staining with CK-20 (purple) or other regions of the pancreas (blue arrow heads). Scale bar, 50 µm. Exenatide did not have any effect on the proliferation of pancreatic ductal cells in diabetic ZDF rats.

CK-20-positive ductal cells were found scattered across the entire tissue and located in main and terminal pancreatic ducts (figure 5). However, proliferative signals (based on Ki67-positive staining) were rarely seen in these cells (figure 5). The percentage of proliferating pancreatic ductal cells was very low (<0.1%) in all groups. There were no exenatide-related effect on ductal cell proliferation rate (0.035 ± 0.050 %, 0.070 ± 0.093 %, 0.063 ± 0.122% for the 6-, 40-, 240-µg/kg/day exenatide doses, respectively) when compared to vehicle control (0.025 ± 0.040%) at the end of the treatment period. The duct proliferation rates at the end of the recovery period remained similarly low and no exenatide-related effects were observed (data not shown).

Apoptotic cells (based on TUNEL positive staining) were observed in all groups, although infrequently, and were scattered throughout the pancreatic tissue. Apoptotic signals were not seen in the ductal CK-20-positive stained cells in pancreata of exenatide-treated animals and they were very rarely seen in ductal cells of control animals. There were no differences in the percent apoptosis parameter for ductal cells of the pancreas of exenatide-treated animals compared to control animals.

## Discussion

The subcutaneous twice-daily administration of exenatide at doses of 6, 40 and 250 µg/kg/day for 3 months in type 2 diabetic obese ZDF rats was associated with expected and beneficial improvements to the disease state with effects on body weight, food consumption, HbA1c, glucose, insulin, minimal-to-moderate hypertrophy of pancreatic islets and survival. Exenatide was considered to be without adverse effect on pancreatic exocrine structure and function, and no exenatide-related effects were observed on pancreatic ductal cell proliferation or apoptosis. Slight increases (within normal ranges) in total amylase concentrations were observed in exenatide-treated animals during the study but were not considered toxicologically significant based on the magnitude of the changes and the absence of any correlating histopathological evidence. Thus, in this study, the no – observed – adverse-effect-level for potential effects on pancreatic exocrine structure and function was determined to be ≥250 µg/kg/day.

The effects of exenatide on glucose metabolism and islet morphology in this study are consistent with other studies performed in diabetic rodents treated with GLP-1RAs 15,22–27. Similar to a previous study in ZDF rats, exenatide-treated animals did not lose weight despite decreased food intake 22. Such dissociation between food intake and weight gain can indicate normalization of metabolic processes with potential augmentation of insulin action and/or with the reversal of glycosuria, as observed in ZDF rats treated with PYY[3–36] 28. Regardless of the mechanism, the disparity between weight gain versus glucose-lowering and insulin stimulation effects indicates that the latter are not being driven by changes in body weight.

Exenatide-treated animals experienced improved survival, reflecting improved condition and disease control. All animals dosed with exenatide survived during the dosing period, with only one animal from the highest-dose group (exenatide 250 µg/kg/day) euthanized during the recovery phase, likely due to an exacerbation of the compromised health status of this animal by study procedures. In contrast, 40% of animals in the control group were euthanized due to deteriorating conditions of diabetes or were found dead during the treatment phase.

Clinical diagnosis of acute pancreatitis in humans is based on severe abdominal pain and radiographically confirmed pancreatic oedema accompanied by increases in amylase and/or lipase at least threefold above the upper limit of normal; lipase concentrations are considered more specific for the diagnosis of pancreatitis than amylase 29. Acinar cells, which secrete digestive enzymes into pancreatic ducts, are considered to play one of the most important roles in the development of pancreatitis; however, mechanisms responsible for developing and modifying the severity of pancreatitis are not well characterized 30. Multiple drugs have been suggested to stimulate or worsen pancreatitis 31.

In this study, absolute or relative (normalized to body weight) pancreatic weights were not affected by treatment with exenatide at all doses tested. No behaviour indicative of abdominal pain or discomfort was reported from daily clinical observations. Histological assessments did not reveal any drug-related structural changes in the exocrine pancreas. Compared to the vehicle control, exenatide treatment did not result in increased lipase concentrations and, moreover, the highest dose of exenatide significantly decreased this enzyme, consistent with previous findings 12. Total amylase concentrations increased in exenatide-treated animals, although this increase was relatively minor and not clinically significant (approximately 30% above values in vehicle-treated rats). Furthermore, amylase concentrations were higher during the study than during the off-drug period in both exenatide-treated and control animals. Amylase continued to increase in control animals over the duration of the study and reached comparable values to exenatide-treated animals at the end of the recovery period.

Changes in pancreatic amylase secretion might be mediated via GLP-1 receptors expressed on acinar cells or via indirect mechanisms. However, in contrast to the unambiguous evidence of the presence of GLP-1 receptors in pancreatic β-cells, the existing literature regarding receptors in acinar cells is very limited and controversial. To our knowledge, there has been no convincing evidence that GLP-1 receptors are expressed in human or rodent acinar cells using validated immunohistochemical approaches complemented with other techniques such as *in situ* ligand binding or *in situ* hybridization. Also, it is not clear whether receptor expression and density is species dependent. GLP-1 receptor expression was seen in ductal cells but was not visible in acinar cells of mouse or rat pancreata 17,24. GLP-1 receptor presence was revealed in acinar cells in some human samples by autoradiography 32 and confirmed by PCR in an acinar cell line; however, GLP-1 did not mediate amylase secretion in these cells 19. Moreover, emerging literature on the development of radiolabelled exenatide analogues for radiotherapy of insulinoma or imaging of β-cell mass in humans would suggest lack of noteworthy GLP-1 receptor expression in any pancreatic cells except β-cells 33,34. Such an argument is supported by our recent (unpublished) observations in rodents using ligand binding and *in situ* hybridization, in which GLP-1 receptor signals were not detected in either acinar or pancreatic ductal cells. Therefore, direct stimulation of acinar cells to secrete digestive enzymes via GLP-1 receptor agonism seems unlikely.

A similar modest increase in pancreatic amylase was reported in a recent study of ZDF rats treated with exenatide and liraglutide 15. Stimulation of amylase secretion might result from paracrine communication between acinar and β-cells; thus, as GLP-1RAs have potent insulinotropic activity, locally increased insulin levels can stimulate insulin receptors in acinar cells leading to enhanced amylase secretion via a well recognized islet-acinar axis 35. The present histological findings do not support the recently postulated hypothesis that increased pancreatic enzyme secretion can be caused by abnormally proliferating and obstructed pancreatic ducts 36. The thorough histological examination did not reveal treatment-related pathological changes in the exocrine pancreas of ZDF rats in the current study, similar to previously published data in other rodent models 12. Furthermore, as confirmed by detailed morphometic analysis, exenatide did not affect apoptosis of ductal cells and their proliferation rate was relatively low and comparable to the proliferation rate in normal human pancreatic ducts 37. Similar to this study, there were no adverse effects on pancreas structure seen in exenatide- and liraglutide-treated ZDF rats 14,15. Additionally, no modification of susceptibility to or severity of experimental pancreatitis was observed in mice treated with exenatide 13.

Other studies do not concur with the present results. Nachnani et al. 16 observed that exenatide did not change amylase but moderately increased lipase in normal rats after chronic treatment (75 days) and caused a subtle increase in acinar inflammation and pyknotic nuclei in the pancreas. Gier et al. 17 reported that chronic activation of GLP-1 receptor by exenatide induced expansion of pancreatic duct glands in normal rats without evidence of pancreatitis. There is some evidence that GLP-1RAs can enhance differentiation of ductal cells to β-cells 19,24,38; therefore, local increases in ductal cell proliferation may also be interpreted as beneficial effects in pancreatic tissue.

Recent commentary by Butler et al. 36 raised a concern that chronic exposure to GLP-1RAs may result in chronic asymptomatic pancreatitis. However, studies performed in multiple disease models showed that exenatide did not evoke pancreatitis and in some instances attenuated the condition 12. The current study, which used ZDF rats – commonly considered to be the gold-standard rodent model of human type 2 diabetes – also does not support the hypothesis that chronic GLP-1RA treatment may lead to pancreatitis.

Recently, Vrang et al. 15 published findings in ZDF rats treated with liraglutide and exenatide in which study design, rigorousness of performance of the study under good laboratory practice conditions, results and conclusions were very similar to those contained herein, further supporting our current findings.

The limitations of this study included lack of exploration of the mechanisms involved in any observed pancreatic exocrine changes. However, beyond the expected metabolic and survival improvements, there were no changes apart from a small increase in plasma amylase, and thus, little reason to study potential mechanisms. Further, we cannot conclude that the source of increased amylase was derived from the pancreas as there was a potential contribution of salivary amylase. Additionally, proliferation rate was generally evaluated in ductal cells identified by positive staining with CK-20 without additional analysis of ductal cell subpopulations in specific ductal structures or in different regions of the pancreas.

In conclusion, long-term exposure to exenatide at plasma concentrations several-fold higher than therapeutic levels in humans resulted in the expected metabolic benefits in the ZDF rat model of type 2 diabetes. Importantly, a significant increase in animal survival was observed with exenatide treatment. Exenatide did not adversely affect exocrine pancreas morphology or function in this model. These data continue to build upon the extensive evidence in animal models showing that exenatide treatment does not cause histopathological changes in the exocrine pancreas that could be associated with pancreatitis or pancreatic malignancies. As nonclinical data may not be fully predictive of human safety, and in the context of a generally very low incidence of pancreatitis, further research is warranted to evaluate whether GLP-1RA-based therapies could be associated with the potential risk for this condition.
